# Collargol Pyelography: A Warning

**Published:** 1914-07-25

**Authors:** 


### RESEARCH AND REMEDIES.

### Collargol Pyelography: A Warning.

There is no doubt that the use of collargol, injected through a ureteric catheter into the pelvis of the kidney, and then photographed by $x$-rays, has proved of enormous assistance to surgeons in the diagnosis of kidney diseases. When the method was first heard of (from America) there were some who raised the theoretical objection that the use of collargol in this manner might not be free from ill-effects. To these objectors it was replied that no such results do, in fact, follow, provided the injection is made properly, the $x$-ray examination done quickly, and the collargol allowed to escape immediately thereafter. Dr. C. W. Vest sounds a note of warning in the *Johns Hopkins Hospital Bulletin*, from which it may be inferred that the method, though fairly safe in expert hands, is not so completely devoid of risk as some of its champions maintain. He makes it clear that due precautions were adopted to avoid accidents, and his conclusions, which are as follows, seem to be justified. In five cases at the Johns Hopkins clinic collargol has been found at operation in the kidney and perirenal tissues; and in one case actually in the peritoneal cavity. The time between the injection and the operation varied from one to five days; one retroperitoneal abscess developed in the series. Both 10-per-cent. and 15-per-cent. solutions were used; no difference in the resulting clinical symptoms was noted. Pain may be quite severe, lasting for two or three days, or even more; Dr. Vest speaks of about one case in five exhibiting this symptom. There may be a definite rise of temperature attributable to the collargol injection. No case of actual nephritis developed, but urinary changes, including albuminuria, are not unknown. In three cases death followed the use of collargol, though it is not considered that the collargol was responsible. Collargol should be used only when absolutely necessary; an alternative medium which will assist diagnosis as well, but be free from ill-effects, is desiderated. Professor Kelly has been experimenting for some time with silver iodide emulsion, and it is thought that possibly this will prove to be superior to collargol. For his candour in recording his unfavourable experiences Dr. Vest is much to be commended.

